# Preliminary study based on methylation and transcriptome gene sequencing of lncRNAs and immune infiltration in hypopharyngeal carcinoma

**DOI:** 10.3389/fonc.2023.1117622

**Published:** 2023-04-25

**Authors:** Kainan Wu, Fen Chang, Wenming Li, Dongmin Wei, Shengda Cao, Yulin Xie, Ce Li, Dapeng Lei

**Affiliations:** ^1^ Department of Otorhinolaryngology, Qilu Hospital of Shandong University, NHC Key Laboratory of Otorhinolaryngology (Shandong University), Jinan, Shandong, China; ^2^ Cheeloo College of Medicine, Shandong University, Jinan, Shandong, China

**Keywords:** hypopharyngeal carcinoma, MeRIP-seq, transcriptome sequencing, lncRNAs, miRNAs, immune cell infiltration

## Abstract

**Background:**

Hypopharyngeal squamous cell cancer (HSCC) is one of the most malignant tumors of the head and neck. It is not easy to detect in the early stage due to its hidden location; thus, lymph node metastasis is highly likely at diagnosis, leading to a poor prognosis. It is believed that epigenetic modification is related to cancer invasion and metastasis. However, the role of m6A-related lncRNA in the tumor microenvironment (TME) of HSCC remains unclear.

**Methods:**

The whole transcriptome and methylation sequencing of 5 pairs of HSCC tissues and adjacent tissues were performed to identify the methylation and transcriptome profiles of lncRNAs. The biological significance of lncRNAs differentially expressing the m6A peak was analyzed by Gene Ontology and Kyoto Encyclopedia of Genes and Genomes. By constructing an m6A lncRNA-microRNA network, the mechanism of m6A lncRNAs in HSCC was analyzed. The relative expression levels of selected lncRNAs were examined by quantitative polymerase chain reaction. The CIBERSORT algorithm was used to evaluate the relative proportion of immune cell infiltration in HSCC and paracancerous tissues.

**Results:**

Based on an in-depth analysis of the sequencing results, 14413 differentially expressed lncRNAs were revealed, including 7329 up-regulated and 7084 down-regulated lncRNAs. Additionally, 4542 up-methylated and 2253 down-methylated lncRNAs were detected. We demonstrated methylation patterns and gene expression profiles of lncRNAs of HSCC transcriptome. In the intersection analysis of lncRNAs and methylated lncRNAs, 51 lncRNAs with up-regulated transcriptome and methylation and 40 lncRNAs with down-regulated transcriptome and methylation were screened, and significantly differentiated lncRNAs were further studied. In the immune cell infiltration analysis, B cell memory was significantly elevated in cancer tissue, while γδT cell amount was significantly decreased.

**Conclusion:**

m6A modification of lncRNAs might be involved in HSCC pathogenesis. Infiltration of immune cells in HSCC might provide a new direction for its treatment. This study provides new insights for exploring the possible HSCC pathogenesis and searching for new potential therapeutic targets.

## 1 Introduction

Hypopharyngeal carcinoma is one of the most malignant head and neck tumors with the worst prognosis. Hypopharyngeal squamous cell carcinoma (HSCC) is the most common pathological type. The incidence of head and neck cancer is about 3%, and its incidence in Asia is about 12.5/100 000, which is 3–4 times higher than that in Europe and America (1). The location of the disease is hidden for examination. With inconspicuous early symptoms, there is a tendency to local infiltration, expansion, and cervical lymph node metastasis in HSCC, making the adjuvant radiotherapy and chemotherapeutic treatment not ideal (2). High postoperative recurrence and metastasis rates are the main reasons for the poor clinical prognosis (3). Dysfunction of pronunciation, breathing, swallowing, neck, and shoulder caused by surgery seriously affects the quality of patients’ life (4, 5). It is of great clinical significance to explore new biomarkers for the occurrence, progression, and prognosis of hypopharyngeal cancer to clarify the mechanism of HSCC occurrence and development and find therapeutic targets for HSCC, which will allow us to improve early diagnosis and prognosis of patients with HSCC.

N-methyladenosine is the most common internal modification in eukaryotic RNA, which regulates RNA stability and translation (6, 7). This dynamic modification is regulated by three proteins called writer, reader, and eraser. Writer comprises a number of proteins, including METTL3, METTL14, and WTAP, which are responsible for RNA methylation (8). Eraser includes m6A RNA demethylase ALKBH5 (9) and obesity-related protein FTO (10) to remove m6A modifications. Reader, which is mainly composed of the YTH-containing domain family (YTHDF1-3 and YTHDC1-2 (11) and insulin-like growth factor-2 mRNA binding protein (IGF2BP2/3) (12), is involved in various steps of RNA metabolism, such as stability, translation, nuclear export, and mRNA splicing. At present, a large number of studies have proven that methylation modification in mRNA plays an important role in autophagy, cancer progression, metabolism, and other processes (13–15).

lncRNA is a class of non-protein-coding RNAs with a length of 200 nucleotides that play key roles in biological processes, such as chromatin progression, transcription, and posttranscriptional modification (16). In terms of mechanism research, lncRNAs often serve as sponges for microRNAs (miRNAs) (17–19). The latest data shows the regulatory mechanism of m6A-modified lncRNAs in cancer (20), which also indicates that m6A-modified lncRNAs might serve as potential biomarkers and therapeutic targets for human cancer. For instance, FTO promotes epithelial-mesenchymal transition (EMT) and inflammatory responses in kidney cells by reducing m6A modification of lncRNAs GAS5 (21). Yang Guo et al. have verified that Linc00520, as the ceRNA of miR-577, promotes the invasiveness of breast cancer in an m6A-dependent manner (22). LncLCAT1 prevents autolysis of IGF2BP2 and promotes lung cancer progression by stabilizing CDC6 mRNA in an m6A-dependent way (23). Additionally, the abnormal expression of lncRNA genes is considered to be involved in the regulation of immune escape (24–26).

There is no report describing the m6A modification of lncRNA in hypopharyngeal carcinoma yet, and no description of immune cell infiltration in hypopharyngeal cancer has been reported. In this study, the expression profile of m6A lncRNAs in HSCC was investigated by m6A-modified RNA immunoprecipitation sequencing (MeRIP-seq). The biological significance of RNAs with differentially expressed m6A peaks by gene ontology (GO) analysis was evaluated and analyzed to explore the possible mechanism by constructing the m6A lncRNA-miRNA network. The infiltrating distribution and differential expression of immune cells in hypopharyngeal carcinoma and adjacent tissues were also demonstrated to provide the basis for further study of immunotherapy in hypopharyngeal cancer.

## 2 Materials and methods

### 2.1 Tissue samples

Five pairs of matched samples of postoperative malignant tumors and adjacent normal hypopharyngeal tissues were confirmed by pathology from five patients who underwent hypopharyngeal cancer resection at Qilu Hospital of Shandong University. Exclusion criteria: 1) non-squamous cell carcinoma, such as adenocarcinoma, malignant lymphoma and soft tissue sarcoma; 2) with other tumors; 3) patients with other acquired, congenital immune deficiency diseases, severe liver and kidney diseases and other systemic diseases; 4) Patients who received preoperative chemotherapy or radiotherapy. All procedures were conducted in accordance with the relevant regulations of the Ethics Committee of Qilu Hospital of Shandong University. Immediately after surgery, 5 pairs of specimens were frozen in liquid nitrogen and stored at −80°C. None of the patients selected for this study had received preoperative chemotherapy or radiotherapy. This study was approved by the Ethics Committee of Qilu Hospital of Shandong University (the ethics approval number is KYLL-2020(KS)-320), and written informed consent was obtained from all participants prior to inclusion. The baseline data of the patients are shown in **Supplementary Table 1**.

### 2.2 Histological analysis of hypopharynx

**Figure 1:**
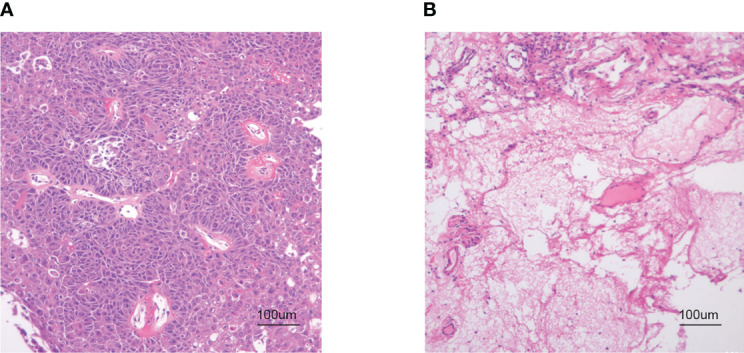
HE staining. **(A)** HSCC carcinoma tissue. **(B)** Hypopharyngeal adjacent tissue (× 100 magnification).

The hypopharyngeal tissue was fixed in a 4% paraformaldehyde solution. After dehydration with ethanol, the tissue samples were embedded in paraffin. The specimens were then cut into sections approximately 4 μm in thickness and stained with hematoxylin and eosin. The slides were examined with a × 100x light microscope (**Figure 1**).

### 2.3 RNA isolation and quality control

TRIZOL reagent (Invitrogen, Carlsbad, Ca, U.S.) was used to extract the total RNA of cancer and cancer-adjacent tissues homogenate. Nano Drop ND - 2000 instrument (Thermo Fisher Scientific, Waltham, Ma, U.S.) was used to measure the concentration of RNA at 280 and 260 optical density (OD). RNA integrity was assessed by denaturing agarose gel electrophoresis. The OD A260/A280 ratio between 1.8 and 2.0 was the standard for RNA purity.

### 2.4 Library preparation and MeRIP-seq

The rRNA Depletion Kit (New England Biolabs, Inc., Massachusetts, USA) was used to remove rRNA from total RNA. Quantitative polymerase chain reaction (qPCR) was used to determine the residual amount of 28S and 18S rRNA, and the removal effect of rRNA was studied. According to the manufacturer’s instructions, m6A RNA immunoprecipitation was performed using the GenSeqTM m6A RNA IP Kit (GenSeq Inc., China). The input samples without immunoprecipitation and m6A IP samples were both used for RNA-seq library generation using the NEBNext^®^ Ultra II Directional RNA Library Prep Kit (New England Biolabs, Inc., USA). Library quality was evaluated by using the BioAnalyzer 2100 system (Agilent Technologies, Inc., USA). The library was sequenced on illumina Hiseq instrument with 150-bp paired-end reads.

### 2.5 MeRIP-seq data analysis

Paired-end reads were obtained from Illumina NovaSeq 6000 sequencer, and their quality was controlled by Q30. After 3’ adaptor-trimming and low-quality read removal by using cutadapt software (v1.9.3), clean reads of all libraries were aligned to the reference genome using Hisat2 software (v2.0.4). MACS software was used to identify methylation sites (peaks) on RNAs. Differentially methylated sites were identified by diffReps. These peaks identified by the software overlapping with exons of lncRNA were figured out and chosen by homemade scripts. Differentially methylated lncRNA-related genes were used for GO and pathway enrichment analysis. GO analysis consisted of three parts, namely biological process (BP) analysis, molecular function (MF) analysis, and cell component (CC) analysis. GO analysis was performed by using the R TOPGO package. The enrichment of each pathway was calculated by Fisher’s exact test in MATLAB MCR software. Bubble chart and columns in figures were used to generate the R package ggplot2 (https://ggplot2.tidyverse.org).

### 2.6 Analysis of whole transcriptome sequencing data

As previously mentioned, after the paired ends were processed by cut adapt software (v1.9.3), high-quality trimmed reads were used to analyze lncRNAs. High-quality reads were aligned with the human reference genome (UCSC hg19) using hisat2 software (v2.0.4). Then, HTSeq software (v0.9.1) was used to obtain a raw count of the transcription level (lncRNA) as the expression profile analysis, and edgeR software (v3.16.5) was used for normalization. Differentially expressed lncRNAs were identified by P-value and fold change. LncRNA target genes were mapped to predict neighboring genes. GO and pathway analyses were performed for these target genes.

### 2.7 Construction of a lncRNA-miRNA network

lncRNAs containing miRNA binding sites can regulate miRNAs. The lncRNA-miRNA network was constructed by selecting the top three up-regulated and down-regulated lncRNAs according to the m6A level. MiRDB software was used to construct the m6A lncRNA-miRNA network, and Cytoscape was used to display lncRNA-miRNA interactions.

### 2.8 qPCR

Real-time quantitative PCR (RT-PCR) was used to detect the expression of screening genes. This study was performed with a BioRad Opticon 2 qPCR machine (Hercules, California, USA) by using SYBR Green Mix (Tien Biotechnology, Beijing, China) and the following cycling procedures: 10 min at 95°C, 40 cycles of 25 s at 95°C, and 1 min at 60°C. β-actin was used as internal control, with each sample running three times.

### 2.9 CCK-8

A Cell Counting Kit-8 (CCK-8, Dojindo, Tokyo, Japan) was used to assess cell viability. Cells transfected with plasmids were cultured in 96-well plates of 10,00 cells per well and cultured for different time periods (0, 24, 48, 72, and 96 hours). Then, 10 mL of CCK-8 solution was added to each well to incubate for 2 hours at 37°C. The OD value of each well was determined by a microplate reader (Bio-Rad) at 450 nm.

### 2.10 Immune cell infiltration

CIBERSORT is a bioinformatics method based on a deconvolution algorithm to estimate the relative proportions of 22 immune cells based on gene expression profiles (27). We used the CIBERSORT package in R with the LM22 gene set from the CIBERSORT website to estimate the total immune infiltration of each sample based on gene expression matrix data. The correlation heat map showed the correlation between the infiltrating immune cells. Histograms were drawn to visualize the infiltration of immune cells in HSCC and paracancerous groups. We also investigated an association of immune infiltrated cells by Spearman correlation coefficient analysis, and a violin chart showed the difference in immune infiltrated proportion between the two groups.

### 2.11 Statistical analysis

GraphPad Prism was used for statistical analysis. Student’s *t*-test was performed to analyze the significance of differences between the groups. The statistical value of all tests was set as P < 0.05.

## 3 Results

### 3.1 Genome-wide methylation analysis of hypopharyngeal cancer patients

**Figure 2:**
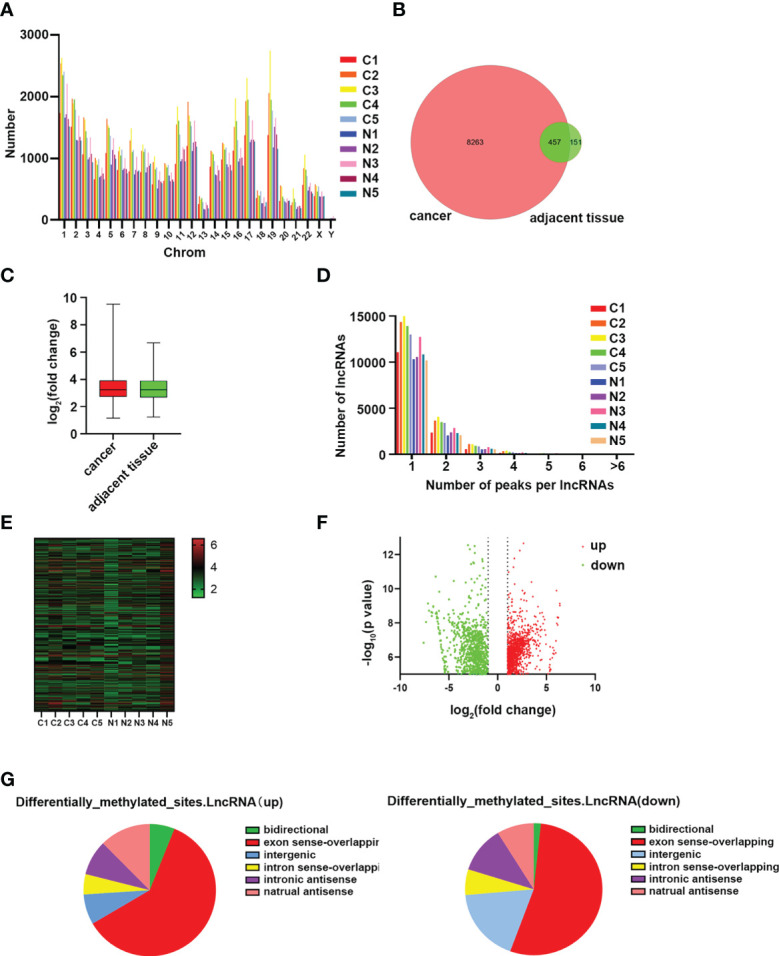
Genome-wide methylation analysis of hypopharyngeal cancer patients. **(A)** Distribution of the number of N6-methyladenosine (m6A) lncRNAs on chromosomes. **(B)** Venn diagram shows the number of specific m6A lncRNAs between the HSCC group and the adjacent tissues group. **(C)** Comparison of m6A peak enrichment levels in lncRNAs between the HSCC group and the adjacent tissues group. **(D)** Statistics of lncRNAs containing different amounts of m6A peaks. **(E)** Hierarchical cluster maps show 5 different m6A modifications in HSCC cancer and adjacent tissues. Higher performance is shown in red, and lower performance is shown in blue. **(F)** Volcanic maps showing lncRNAs with significantly differentially expressed peaks of m6A. **(G)** Genomic origin and distribution of lncRNAs expressing differentiated m6A in HSCC and paracancerous tissues.

The expression of m6A lncRNAs in both cancerous and paracancerous tissues was investigated by using the MeRIP-seq method. Prior to MeRIP-seq, residual rRNA assays at 28S and 18S showed that rRNA was effectively removed from total RNA. Among all chromosomes, chromosome 1 had the most m6A lncRNAs in both cancerous and paracancerous tissues (**Figure 2A**). Among them, 8263 cases were specifically expressed in the HSCC group, and 151 cases were specifically expressed in the adjacent tissues group, including 457 cases shared by the two groups (**Figure 2B**). The m6A level of total lncRNAs in the HSCC group was comparable to that in the adjacent tissues group (**Figure 2C**). In addition, more than 90% of lncRNAs in both HSCC and adjacent tissues groups contained one or two m6A peaks (**Figure 2D**). lncRNAs with differentially expressed m6A peaks were further analyzed to understand the biological role of m6A modification of lncRNAs in HSCC. Significantly different expressions were defined as fold change > 2 and P < 0.00001. Compared with the adjacent tissues group, 5218 lncRNAs with differentiated m6A peaks were identified in the HSCC group, and 4542 cases were up-regulated, while 2253 cases were down-regulated. The top 10 up- and down-regulated methylated m6A sites in lncRNAs are listed in **Table 1**. The expression profiles of m6A lncRNAs in HSCC and adjacent tissues by hierarchical clustering analysis is shown in **Figure 2E**. Scatter plots showed changes in differentiated m6A lncRNAs (**Figure 2F**). The gene origin distribution of m6A differentiated lncRNA is shown in **Figure 2G**.

**Table 1:** The top 10 up- and down-regulated methylated m6A sites in lncRNAs between the HSCC group and the adjacent tissues group.

chrom	txStart	txEnd	PeakiD	transcript_id	GeneName	Foldchangf	P_value	Regulation
chr14	64805141	64805317	diffreps_peak_153162	ENST00000359491	RP11-544120.2	82.1	9.30E-10	up
chr14	64805141	64805317	diffreps_peak_153162	ENST00000359491	RP11-544120.2	82.1	9.30E-10	up
chr11	67791801	67792000	diffreps_peak_89412	ENST00000527514	ALDH3B1	81.3	7.09E-10	up
chr11	67791801	67792000	diffreps_peak_89412	ENST00000527514	ALDH3B1	81.3	7.09E-10	up
chr11	67791801	67792000	diffreps_peak_89412	ENST00000527514	ALDH3B1	81.3	7.09E-10	up
chr11	67791801	67792000	diffreps_peak_89412	ENST00000527514	ALDH3B1	81.3	7.09E-10	up
chr11	67791801	67792000	diffreps_peak_89412	ENST00000527514	ALDH3B1	81.3	7.09E-10	up
chr11	67791801	67792000	diffreps_peak_89412	ENST00000527514	ALDH3B1	81.3	7.09E-10	up
chr14	19854801	19855020	diffreps_peak_144522	ENST00000551334	CTD-2314B22.3	73.8	4.64E-09	up
chr7	155089201	155089251	diffreps_peak_484061	ENST00000609974	AC144652.1	72.4	4.89E-09	up
chr1	152996681	152996736	diffreps_peak_30101	ENST00000429623	RP1-20N18.4	194.95518	1.42E-07	down
chr1	204212241	204212340	diffreps_peak_41384	uc009xau.1	PLEKHA6	164.2	8.58E-09	down
chr1	204212241	204212340	diffreps_peak_41384	uc009xau.1	PLEKHA6	164.2	8.58E-09	down
chrX	139298186	139298578	diffreps_peak_552948	ENST00000458577	AC004070.1	142.7	7.06E-10	down
chr13	19407856	19407989	diffreps_peak_130127	ENST00000457997	ANKRD20A9P	132.4	1.87E-09	down
chr16	77477741	77477880	diffreps_peak_196649	ENST00000564358	RPll-449110.1	112.1	2.28E-09	down
chr10	77036133	77036180	diffreps_peak_64412	ENST00000412453	HMGA1P5	109.1	3.81E-09	down
chr11	134254541	134254920	diffreps_peak_102908	ENST00000531778	B3GAT1	105.3	2.47E-09	down
chr11	134254541	134254920	diffreps_peak_102908	ENST00000531778	B3GAT1	105.3	2.47E-09	down
chr11	134254541	134254920	diffreps_peak_102908	ENST00000531778	B3GAT1	105.3	2.47E-09	down

### 3.2 Functional analysis of lncRNAs with differentiated m6A peak

**Figure 3:**
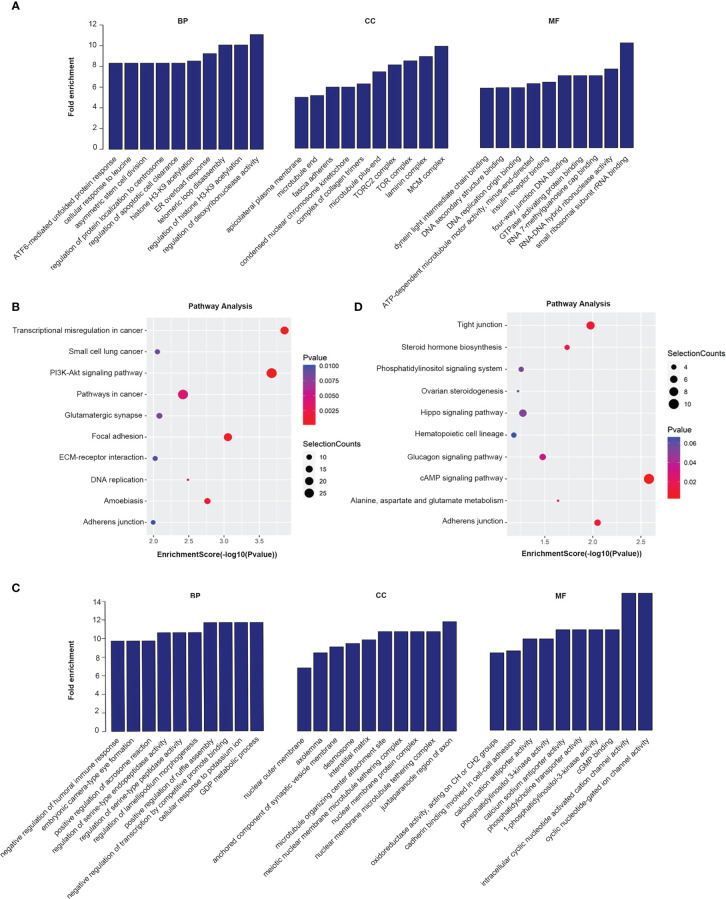
GO and KEGG analysis of parental gene of m6A-related lncRNAs with differentiated peaks. **(A, B)** Analysis of lncRNAs up-regulated by N6-methyladenosine (m6A) peaks by Gene Ontology (GO) and Kyoto Encyclopedia of Genes and Genomes (KEGG). **(C–D)** GO and KEGG analysis of lncRNAs with down-regulated peak m6A.

**Figure 4:**
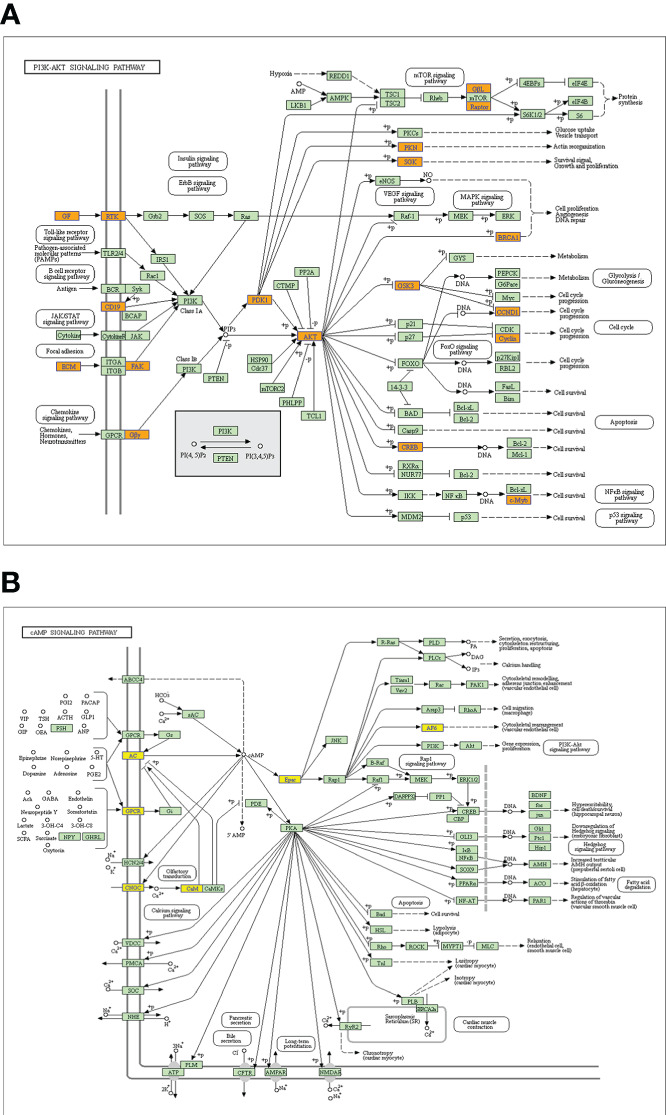
Signaling pathways of a parental gene of m6A-related lncRNAs with differentiated peaks. **(A)** The PI3K-AKT signaling pathway obtained from lncRNA up-regulated by an m6A peak in HSCC progression. **(B)** The cAMP signaling pathway obtained from lncRNA down-regulated by an m6A peak in HSCC progression.

GO analysis and KEGG pathway analysis were performed for lncRNAs containing differentiated m6A peaks to investigate the role of m6A-related lncRNAs in HSCC. The top 10 GO terms of lncRNAs with elevated m6A peaks from the perspectives of BP, CC, and MF are shown in **Figure 3A**. For BP, the most abundant and meaningful GO project was the regulation of deoxyribonuclease activity. For CC, the number 1 spot was the MCM complex. For CC, the most significant one was small ribosomal subunit rRNA binding. KEGG pathway analysis was performed on lncRNAs up-regulated by m6A peak, and the top 10 pathways were selected and shown in the bubble chart (**Figure 3B**). Among them, PI3K-AKT was a major signaling pathway associated with HSCC progression (**Figure 4A**). The GO terms of lncRNAs down-regulated by m6A peak are shown in **Figure 3C**. For BP, the top 4 were GDP metabolic process, cellular response to potassium ion, negative regulation of transcription by competitive power promoter binding, and positive regulation of ruffle assembly. The juxtaparanode region of the axon was the most abundant term for CC. For MF, the main GO terms were cyclic nucleotide-gated ion channel activity and intracellular cyclic nucleotide-activated cation channel activity. For the KEGG pathway analysis of lncRNAs with m6A peak down-regulation (**Figure 3D**), the cAMP signaling pathway was the main pathway (**Figure 4B**).

### 3.3 Changes in the expression of HSCC-related genes

**Figure 5:**
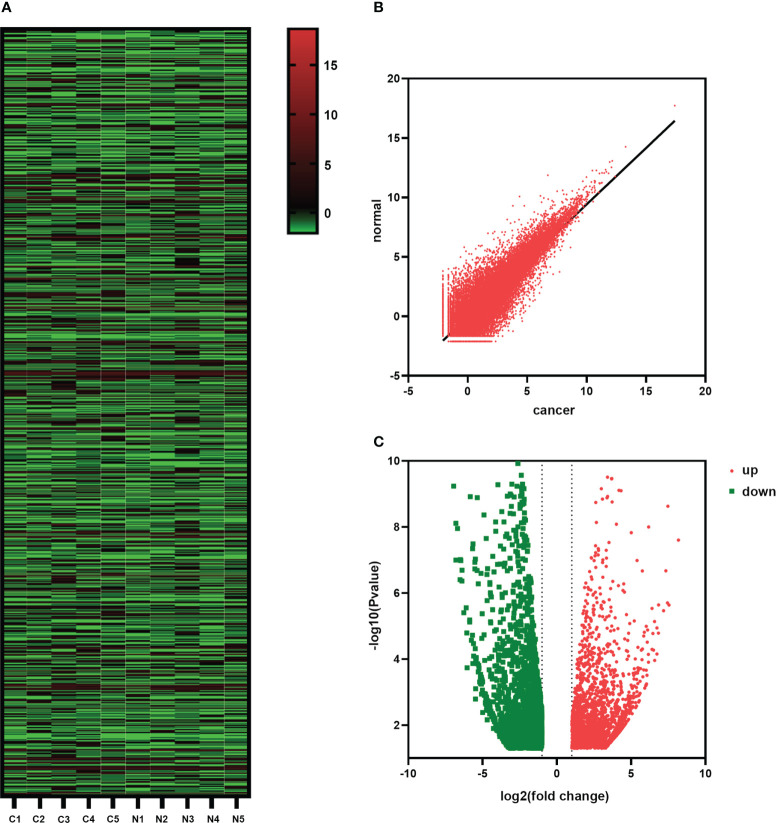
Changes in the expression of HSCC-related genes. **(A)** The hierarchical cluster diagram shows the different expression levels of lncRNAs in HSCC and adjacent tissue groups. Higher performance is shown in red, and lower performance is shown in blue. **(B)** Scatter plots showing lncRNAs with significant differential expression. **(C)** Volcanic shows the overall expression of up-regulated and down-regulated lncRNAs between the HSCC group and paracancerous tissues.

RNA-seq was used to analyze the transcriptome of paired tumors and adjacent normal tissues from 5 pairs of HSCC patients to investigate the changes in HSCC expression patterns. RNA-seq was used to detect gene expression in tumor and paracancerous tissues. Hierarchical clustering analysis was conducted to show the expression profiles of transcriptomes in HSCC and adjacent tissues groups (**Figure 5A**). Scatter plots showed the changes of related differentiated genes between the HSCC group and the adjacent tissues group (**Figure 5B**). Significantly different expressions were defined as fold change > 2 and P < 0.05. Of these, 14413 genes were differentially expressed between tumor tissues and adjacent normal tissues (**Supplementary Table 2**). Among them, 7329 cases were up-regulated, and 7084 cases were down-regulated in the HSCC group. The volcano plot showed the overall expression of up-regulated and down-regulated lncRNAs in the HSCC group and paracancerous tissues (**Figure 5C**).

### 3.4 Screening of HSCC-related differential methylation candidate genes

Genes were screened by the following procedure to identify candidate genes involved in hypopharyngeal cancer. First, in the differential methylation results, p-value < 0.00001 and fold change > 0.2 was the threshold for screening differentially methylated genes. Then, in the differential expression results of whole transcriptome sequencing, p-value < 0.05 and fold change > 2 were the threshold for screening differentially expressed genes. Finally, 51 lncRNAs were obtained by crossing the hypermethylated and hyperexpressed genes. The hypomethylated and underexpressed genes were crossed to obtain 40 lncRNAs (**Supplementary Table 3**). After excluding lncRNAs that were unqualified according to their expression profiles and difficulty to design with primers, methylation fold change was taken as the standard. The top 3 highly methylated and highly expressed lncRNAs (lncDLGAP1-AS2, lncIGF2BP2, and lncFAHD2CP) and the top 3 hypomethylation and lowly expressed lncRNAs (lncAGBL1-AS1, lncGPR78, and lncULK4P3) were selected.

### 3.5 Identification of HSCC-related lncRNAs by qPCR and CCK-8

**Figure 6:**
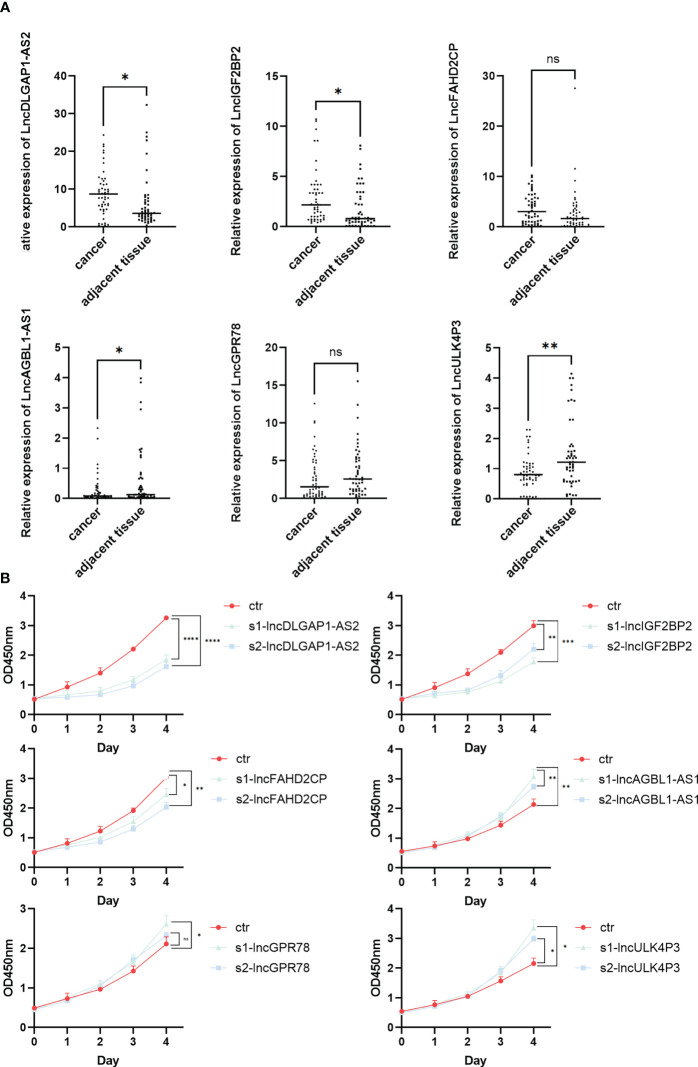
Identification of HSCC-related lncRNAs by qPCR and CCK-8. **(A)** The lncRNA expression level of lncDLGAP1-AS2, lncIGF2BP2, lncFAHD2CP, lncAGBL1- AS1, lncGPR78, and lncULK4P3 in 50 pairs of HSCC and paracancerous tissues quantified using qRT-PCR, *p < 0.05, **p < 0.01. **(B)** OD450 values discount diagram of lncDLGAP1-AS2, lncIGF2BP2, lncFAHD2CP, lncAGBL1-AS1, lncGPR78, and lncULK4P3 in control groups and knockdown groups using CCK-8, *p < 0.05, **p < 0.01, ***p < 0.001, ****p < 0.0001. ns, p>0.05.

The expression levels of the above-screened lncRNAs were verified by qPCR in HSCC and matched paracancerous tissues (n = 50). The results showed that the expressions of lncDLGAP1-AS2, lncIGF2BP2, lncAGBL1-AS1, and lncULK4P3 were significantly different between cancer and paracancerous tissues (**Figure 6A**). Further, we verified the changes at the cellular level. We designed siRNA targeting for these 6 lncRNAs and then conducted a CCK-8 assay. The results showed that the OD450 values of these 6 lncRNAs were different from those of the control group after knockdown (**Figure 6B**; primers and siRNA sequences in **Supplementary Table 4**).

### 3.6 Construction of the m6A lncRNA-miRNA network in HSCC

**Figure 7:**
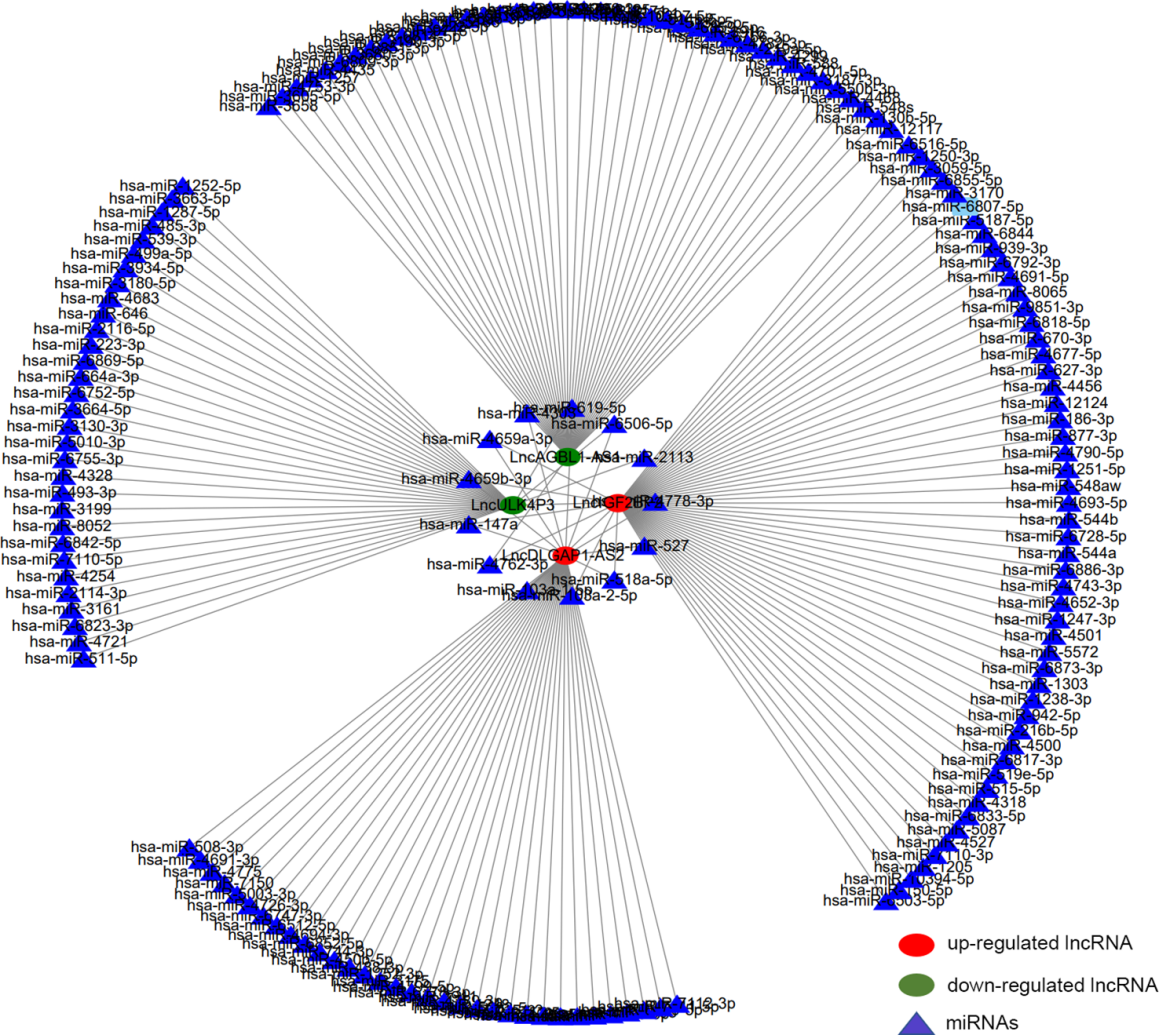
Construction of the m6A lncRNA-miRNA network in HSCC. The network diagram describes 4 interested lncRNAs and about 186 potential binding target miRNAs. Red circles represent hypermethylated lncRNAs, green circles represent hypomethylated lncRNAs, and triangles represent miRNAs.

After tissue and cell level verifications, we further obtained 4 lncRNAs with statistical significance (lncDLGAP1-AS2, lncIGF2BP2, lncAGBL1-AS1, and lncULK4P3). We constructed related lncRNA-miRNA networks to further explore the underlying mechanisms of the genes screened above. In this network diagram, we showed all the miRNAs that may bind to these 4 lncRNAs that were screened (**Supplementary Table 5**). Several important miRNAs interacted with more than one lncRNA (**Figure 7**). We speculate that the binding of miRNAs and lncRNAs might play a role in the occurrence and development of HSCC, which is worth further discussion.

### 3.7 Distribution, correlation, and differential analysis of infiltrated immune cells

**Figure 8:**
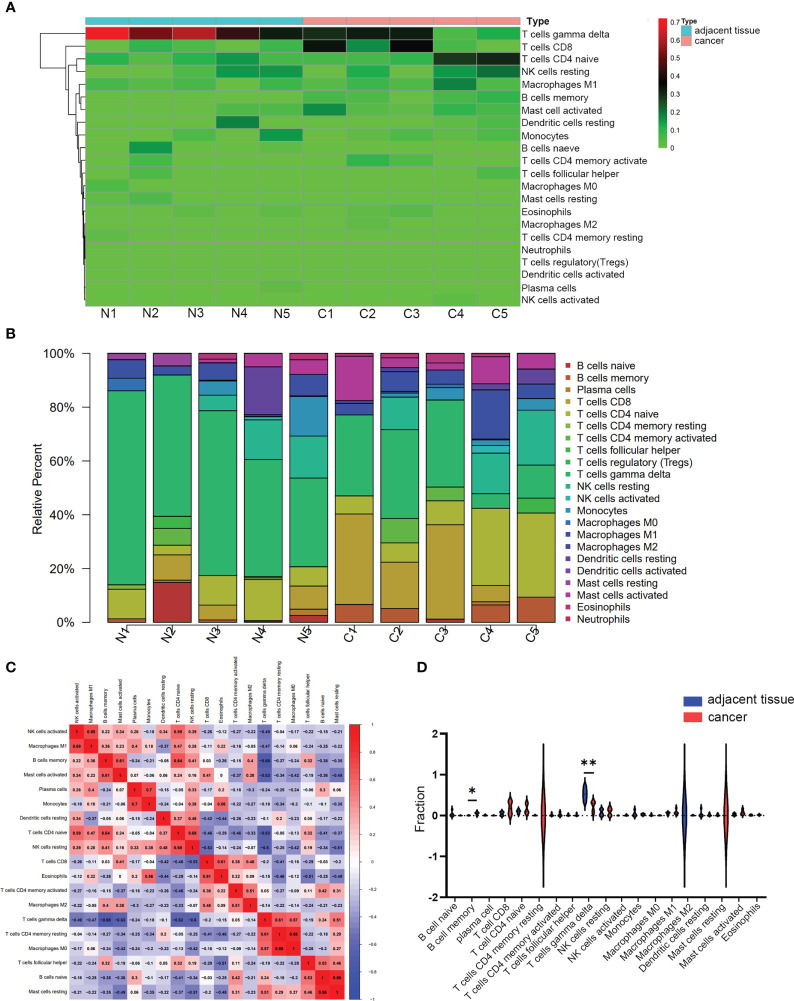
Immune infiltrating analysis. **(A, B)** The relative percentage of 22 subpopulations of immune cells in 5 pairs of HSCC tissues and paracancerous tissues. **(C)** CorHeatmap of 22 subpopulations of immune cells in 5 pairs of HSCC tissues and paracancerous tissues. **(D)** Vioplot of 22 subpopulations of immune cells in 5 pairs of HSCC tissues and paracancerous tissues. *p < 0.05, **p < 0.01.

CIBERSORT inverse convolution method with *p* < 0.05 was used to obtain the heat map of 5 hypopharyngeal carcinoma tissues and 5 paracancerous tissues. γδT cells in paracancerous tissues of HSCC patients were significantly more abundant than that in hypopharyngeal carcinoma tissues, while CD8^+^ T cells and naïve CD4^+^ T cells in cancer tissues were higher than those in paracancerous tissues (**Figure 8A**). **Figure 8B** details the distribution of 22 immune cells in each sample. A positive correlation was detected between macrophage M1 and activated NK cells (r = 0.69), activated mast cells and memory B cells (r = 0.61), monocytes and plasma cells (r = 0.7), naïve CD4^+^ T cells and memory B cells (r = 0.64), resting NK cells and naïve CD4^+^ T cells (r = 0.68), eosinophils and CD8^+^ T cells (r = 0.61), M0 macrophages and resting memory CD4^+^ T cells (r = 0.88), as well as resting mast cells and naïve B cells (r = 0.86). Instead, a negative correlation was detected between γδT cells and memory B cells (r = −0.68), γδT cells and activated mast cells (r = −0.63), γδT cells and naïve CD4^+^ T cells (r = −0.62), as well as γδT cells and resting NK cells (r = −0.6) (**Figure 8C**). Vioplot showed the difference of immune infiltrating cells in HSCC and paracancerous tissues. The number of memory B cells was significantly elevated in cancer tissue, while the number of γδT cells was significantly decreased (**Figure 8D**).

## 4 Discussion

Many studies have identified the role of lncRNAs and m6A modifications in tumorigenesis, tumor progression, and innate immunity (28). M6A modification is the most common and abundant form of RNA modification and plays a crucial role in cancer development by regulating m6A demethylases, methyltransferases, and binding proteins (29). There is a close correlation between m6A regulators and lncRNAs. However, the correlation between m6A modifications of lncRNAs and hypopharyngeal cancer remains unclear.

In this study, m6A-lncRNA in HSCC was studied by MeRIP-seq for the first time. The outcome supports the dynamic characterization of m6A epigenetic modifications in HSCC. The lncRNA transcriptome profile and m6A modification profile in HSCC was identified by using RNA-seq and MeRIP-seq, and its biological significance and potential mechanism were determined. The main findings were as follows (1). The methylation degree of lncRNAs in HSCC tissues and adjacent tissues was similar, but there were many lncRNAs with differentiated m6A modification. A total of 5218 lncRNAs with differentiated m6A peaks were identified. Among them, 3471 cases were up-regulated, and 1747 cases were down-regulated in the HSCC group. These differentiated m6A lncRNAs were involved in key processes of HSCC, such as the PI3K-AKT signaling pathway based on the GO and KEGG analysis (2). Four lncRNAs with different methylation degrees and expressions in HSCC tissues and adjacent tissues were screened and verified by qPCR in tissues and CCK-8 in cell lines, which laid a foundation for the subsequent research (3). The m6A lncRNA-miRNA network was constructed, and we predicted there were many miRNAs involved in the occurrence and development of HSCC, suggesting that m6A might affect the interaction of miRNAs (4). Immune infiltration analysis suggested that the occurrence and development of HSCC were related to immune response. This paper provides a new possibility for finding effective therapeutic targets for HSCC and exploring the potential molecular mechanism of HSCC.

In our transcriptome sequencing results, we found that PI3K-AKT signaling pathway is involved in the progression of HSCC. For the PI3K-AKT signaling pathway analyzed and summarized before, several studies have demonstrated that it is related to the regulation of m6A modification. For example, Wen-Jing Gong et al. have demonstrated that METTL3 promoted apoptosis, autophagy, and pyroptosis of glutamic acid-induced intrahepatic cholangiocarcinoma (ICC) by interacting with DGCR8 and modulating the miR-30b-5p/PIK3R2 axis in an m6A-dependent manner (30). METTL3 down-regulation attenuates fusarium-reduced corneal inflammation *via* PI3K/AKT signaling in mice (31). METTL3 promotes PI3K expression by introducing m6A modifications in the 3’ untranslated region (3’UTR) of PI3K. Elevated PI3K levels activate downstream AKT and mTOR signaling pathways, leading to rapid proliferation and metastasis of lung cancer cells (32). Also, the lncRNAs screened in this study have also been reported in the literature. DLGAP1-AS2 was significantly increased in colorectal cancer (CRC) tissues and cell lines, and silencing DLGAP1-AS2 significantly decreased Myc mRNA and protein levels. Blocking Myc effectively abrogated the enhanced invasive behavior of CRC cells induced by DLGAP1-AS2 overexpression (33). Our study still needs to further prove the specific mechanism of lncRNAs in PI3K-AKT signaling pathway in HSCC.

Immune cells are an important part of the tumor microenvironment (TME). The relevance of immune cells to tumor development and treatment has been increasingly recognized (34). Our study mapped the immunoinfiltration profile of HSCCS based on transcriptome sequencing of lncRNAs. In this study, memory B cells were significantly increased in the HSCC group; hence, it is speculated that immune response plays an important role in HSCC occurrence and progression. Existing studies have found that the abnormal expression of lncRNA is closely related to the pathogenesis of various diseases. lncRNA is involved in cell migration (35) and immune tolerance by regulating the development and apoptosis of immune cells (36). We expect to find further evidence to prove that the methylation-related lncRNAs screened above participate in the immune regulation of HSCC and, thus, affect the progression of hypopharyngeal cancer.

Admittedly, the study had some limitations. First, the mechanism of these m6A-related lncRNAs was not elucidated enough, and the miRNAs interacting with them were not fully verified. Their role in affecting TME is still unclear and needs to be further studied. In addition, a larger sample size is required to further verify the expression and role of lncRNA. Unfortunately, this study was based on tumor samples obtained after patients had undergone invasive surgery and could not be further studied in body fluids. Despite these limitations, m6A-related lncRNAs and successfully screened-out lncRNAs were identified in this study, which might play a key role in HSCC progression and prognosis.

In conclusion, a total of 6 m6A-related lncRNAs were screened from the whole transcriptome and methylation sequencing. Then, the expression levels of these 6 lncRNAs in HSCC and adjacent tissues were verified by qPCR. Two lncRNAs with high methylation and high expression level and two lncRNAs with low methylation and low expression level were finally identified and the miRNAs bound by these 4 lncRNAs were predicted. Key insights into the occurrence and progression of m6A-related lncRNAs in HSCC were provided based on the results of this study.

## Data availability statement

The data presented in the study are deposited in the Dryad repository, accession number https://doi.org/10.5061/dryad.g1jwstqvs.

## Ethics statement

The studies involving human participants were reviewed and approved by Medical Ethics Committee of Qilu Hospital of Shandong University. The patients/participants provided their written informed consent to participate in this study.

## Author contributions

DL and FC conceived and designed the experiments; KW performed the experiments and wrote the manuscript; FC, WL, DW, SC, YX, and CL provided critical comments and suggestions, and FC revised the manuscript. All authors contributed to the article and approved the submitted version.
